# Aquaporin-4 antibody-positive myelitis initially biopsied for suspected spinal cord tumors: Diagnostic considerations

**DOI:** 10.1177/1352458513505350

**Published:** 2013-09-12

**Authors:** Douglas Kazutoshi Sato, Tatsuro Misu, Cristiane Franklin Rocha, Dagoberto Callegaro, Ichiro Nakashima, Masashi Aoki, Kazuo Fujihara, Marco Aurelio Lana-Peixoto

**Affiliations:** 1 Department of Neurology, Tohoku University Graduate School of Medicine, Japan; 2 Department of Multiple Sclerosis Therapeutics, Tohoku University Graduate School of Medicine, Japan; 3 CIEM MS Research Center, Federal University of Minas Gerais Medical School, Brazil; 4 Department of Neurology, Faculty of Medicine, University of Sao Paulo, Brazil

**Keywords:** Neuromyelitis optica, spinal cord tumor, biopsy, differential diagnosis, pathology, aquaporin-4

## Abstract

Two patients with longitudinally extensive myelopathy were initially biopsied for suspected spinal cord tumors. Both patients were later diagnosed with neuromyelitis optica spectrum disorders (NMOSD) supported by their AQP4-seropositivity. Pathological review of both biopsies revealed demyelinated lesions with thickened vessel walls and tissue rarefaction. Immunohistochemical staining demonstrated findings compatible with acute NMOSD lesions in one case while the other case exhibited findings consistent with chronic NMOSD lesions. A pre-biopsy differential diagnosis of longitudinally extensive spinal cord tumors should include NMOSD. Specific biopsy features, such as cystic changes with vascular wall thickening and astrocyte injury, should raise suspicion for NMOSD.

## Introduction

Longitudinally extensive spinal cord lesions on magnetic resonance imaging (MRI) are observed in a variety of diseases affecting the spinal cord, such as spinal cord tumors, inflammation, compressive myelopathy, infections, arteriovenous malformations and ischemia.^1^ In some cases, the diagnosis can be made only by pathological analysis, despite the risks of surgical complications and sequelae.

Longitudinally extensive transverse myelitis (LETM) stretching over three or more vertebral segments on MRI is commonly seen in neuromyelitis optica spectrum disorders (NMOSD).^2^ The presence of antibodies against aquaporin-4 (AQP4) is an important diagnostic biomarker of NMOSD, but no assay is 100% sensitive, and the test is unavailable in some regions.^3^ AQP4 antibody positivity is crucial because immunosuppressive therapy is effective in reducing further attacks in NMOSD.^4^

Here, we report the clinical, imaging and pathological features of two patients with LETM (without optic neuritis attacks) and AQP4 antibodies by tissue-based indirect immunofluorescence^5^ who initially underwent spinal cord biopsies for suspected tumors.

## Case reports

### Case 1

**Figure 1.**
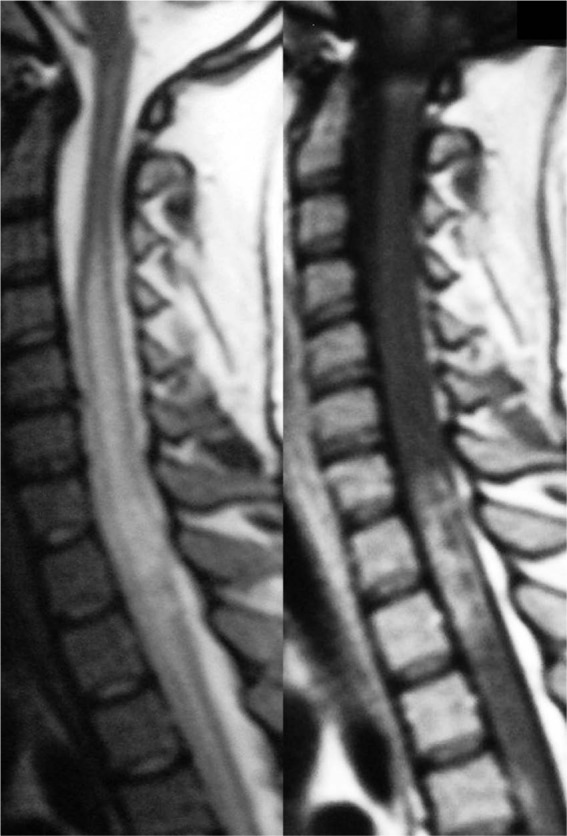
Sagittal T2- (left) and T1-weighted (right) magnetic resonance imaging (MRI) of the cervical spinal cord from case 1 shows a longitudinally extensive, centrally located cord lesion extending from the cervical (C3) to the thoracic cord (T4) with gadolinium contrast enhancement during the second attack. The patient also had a second nonenhancing lesion in the lower thoracic cord (T9–T12; not shown).

A 14-year-old girl presented with bilateral lower limb paresthesia and weakness that progressed over a few days. Nine months later, she developed upper extremity weakness with sensory dysfunction at the level of C7 that worsened in a step-wise manner over a period of 3 months to paraplegia, sensory loss and sphincter dysfunction. Spinal MRI (Figure 1) showed tumefactive, T2 high-intensity lesions from C3 to T4 (with contrast enhancement) and T9 to T12 (without contrast enhancement). Brain MRI revealed only a few nonspecific cerebral white matter lesions. She then underwent thoracic spinal cord biopsy for suspicion for a spinal astrocytoma. However, the initial pathological analysis excluded tumor, and no definite diagnosis was made. Later, her serum AQP4 antibody returned as positive, and she was diagnosed with NMOSD. Cerebrospinal fluid (CSF) analysis revealed mild pleocytosis with 17 cells/mm^3^ (12% neutrophils), and an increased protein level of 81.4 mg/dl. No clinical response was observed with high-dose methylprednisolone.

**Figure 2.**
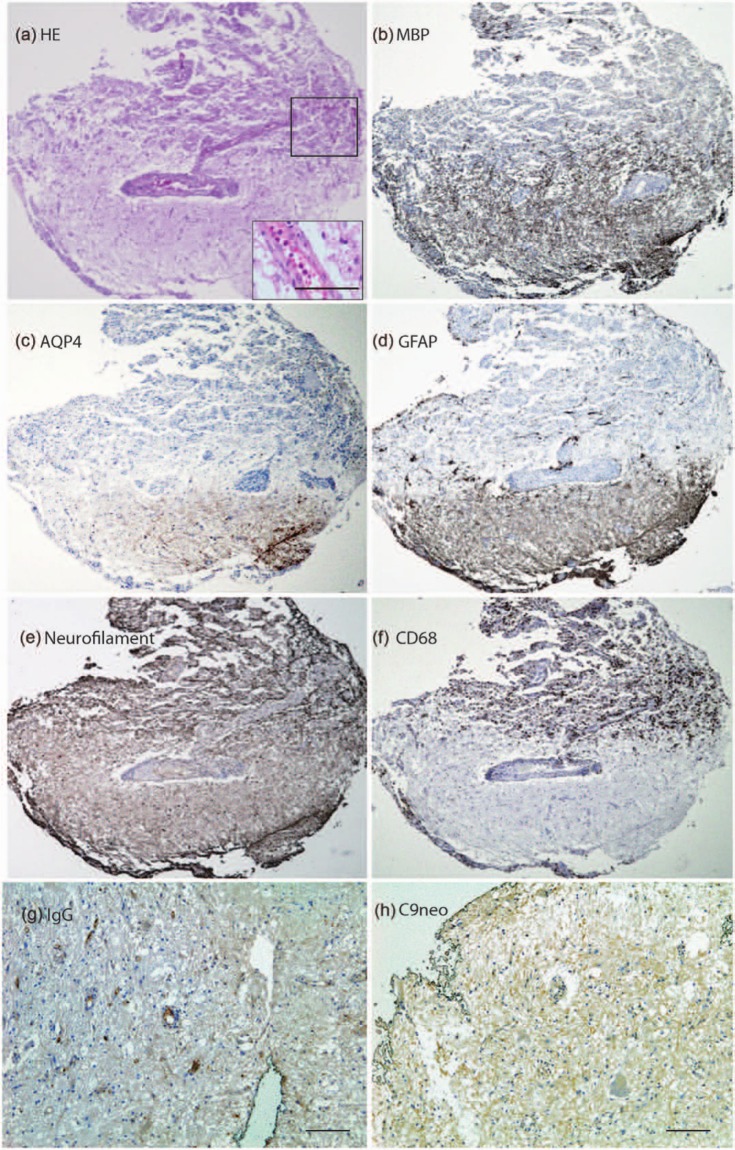
Pathological findings compatible with acute neuromyelitis optica spectrum disorders (NMOSD) in serial sections from case 1. (a) Acute inflammatory cystic lesions with hyalinized or thickened vessel walls (predominantly in the upper two-thirds of the specimen). Perivascular infiltration with mononuclear and polymorphonuclear cells, including eosinophils (detail in mag 200×). Loss of staining for (b) myelin basic protein (MBP), (c) aquaporin-4 (AQP4) and (d) glial fibrillary acidic protein (GFAP). AQP4 loss is more extensive than GFAP loss, and MBP is relatively preserved compared to GFAP and AQP4 (lower third of the specimen). (e) Neurofilament staining is relatively preserved even in areas with active inflammation ((a)–(e), mag 40×). The lesions (f) exhibit remarkable CD68-positive macrophage infiltration with (g) star-shaped or microvenule staining pattern of immunoglobulin G (IgG) and (h) activated complement deposition (C9neo). The images (g) and (h) show higher magnifications (100×) of the area shown in (a). Bar scale: 100 µm.

Re-assessment of the biopsied spinal cord specimen (Figure 2) revealed massive necrotic areas with prominent cellular infiltration, cystic inflammatory lesions with hyalinized or thickened vessel walls and astrocyte rarefaction. In perivascular areas, we observed inflammatory cells including eosinophils, neutrophils and lymphocytes. Immunohistochemistry revealed the AQP4 immunoreactivity loss to be more extensive than that of glial fibrillary acidic protein (GFAP), whereas myelin basic protein (MBP)-stained myelinated fibers were relatively preserved. The cellular infiltrates in the severely affected area included CD68-positive macrophages. The deposition of immunoglobulin G (IgG) and C9neo complement was present at the astrocytic foot processes and microvenules, but there was no typical rosette-pattern of C9neo deposition. Moreover, neurofilament-positive axons were relatively spared even in lesions demonstrating a complete loss of AQP4 and GFAP.

### Case 2

**Figure 3.**
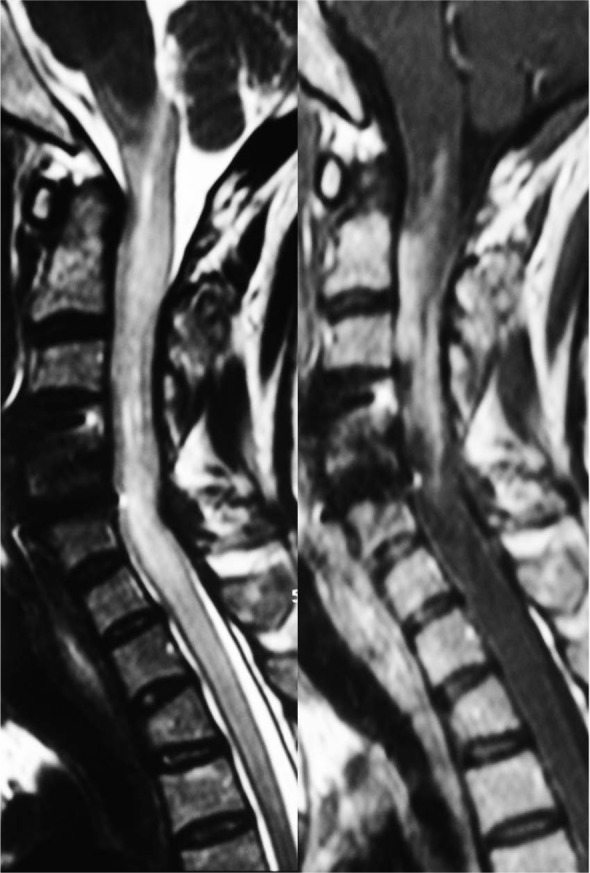
Sagittal T2- (left) and T1-weighted (right) magnetic resonance imaging (MRI) of the cervical spinal cord from case 2 shows a longitudinally extensive, edematous, enhancing, centrally located cervicothoracic cord lesion extending into the medullary region, during the second myelitis attack.

A 47-year-old woman presented with left shoulder pain, motor weakness and paroxysmal spasms in her left upper extremity over a month period. Spinal cord MRI demonstrated an edematous T2 hyper-intense lesion extending from the medulla to C7 with central contrast enhancement. Brain MRI was normal. She was treated with high-dose methylprednisolone for five days with a partial recovery. Four months later, acute transverse myelopathy developed over a few days with paraparesis, loss of sensation in both legs and sphincter dysfunction. A second spinal MRI revealed a caudal extension of the lesion to the T2 level with marked swelling and gadolinium enhancement (Figure 3). The patient underwent a cervical cord biopsy based on a presumptive diagnosis of spinal astrocytoma, but the initial pathology showed no evidence of tumor. Further investigation revealed that her serum AQP4 antibody was positive.

**Figure 4.**
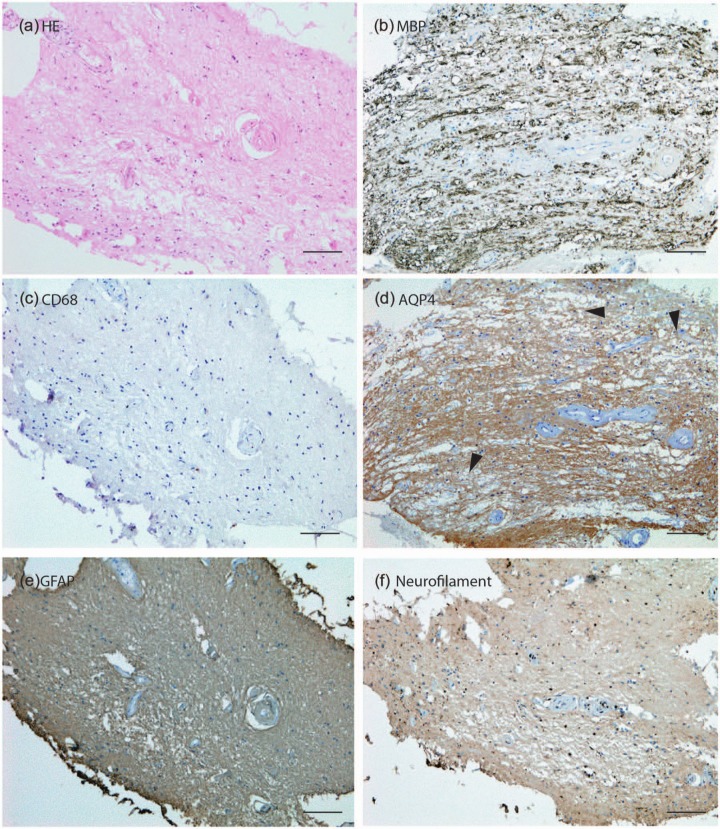
Pathological findings in serial sections from case 2 compatible with chronic neuromyelitis optica spectrum disorders (NMOSD) lesions. (a) Lesion in the chronic stage demonstrating cystic changes and vascular hyalinization without any inflammatory cell infiltration. (b) MBP-stained myelin fibers or axons were observed with thinly re-myelinated fibers but (c) without actively myelin-laden CD68+ macrophages. (d) Demyelinated lesions had high levels of aquaporin-4 (AQP4) expression suggestive of reactive fibrous astrogliosis, in addition to speckled necrotic spots with decreased AQP4 staining (arrows). (e) Glial fibrillary acidic protein (GFAP) staining is diffusely increased suggesting reactive astrogliosis. (f) Neurofilament staining shows some areas with rarefaction suggesting previous tissue injury. Bar scale: 100 µm.

Re-assessment of the spinal cord specimen (Figure 4) revealed some cystic changes in the tissues, vascular wall thickening and hyalinization, but no inflammatory cell infiltration. Immunohistochemistry demonstrated the demyelinated lesions to be mostly covered by fibrous astrogliosis that stained strongly for AQP4. MBP-positive myelinated fibers were partially observed. The lesion also contained some cystic necrotic spots with losses of AQP4 immunoreactivity.

## Discussion

These two case reports highlight the importance of a proper diagnostic work-up in patients with acute LETM to differentiate inflammatory myelitis from spinal tumors. Serum testing for AQP4 antibodies should be considered before invasive procedures.

Most of our understanding of NMOSD pathology derives from post-mortem studies.^6,7^ Spinal cord-biopsied lesions in patients with NMOSD are uncommon for obvious reasons, and the opportunity to evaluate NMOSD pathology features in vivo is rare. We identified only one prior case report of a biopsied patient with LETM and AQP4 antibodies, and these authors did not perform a detailed pathological study to analyze features of NMOSD.^8^

Acute NMOSD lesions are characterized by an extensive loss of AQP4 and GFAP staining with activated complement and IgG deposition in perivascular regions, in addition to relatively preserved myelinated fibers. In regions with severe inflammation, necrotic changes and perivascular cuffing are observed, predominantly including neutrophils, eosinophils, lymphocytes and macrophages.^6,7^ NMOSD findings are quite distinct from multiple sclerosis (MS) lesions and clearly indicate immune-mediated astrocytopathy.^6,9^ The pathological findings in the first case are good examples of the active NMO lesions associated with complement activation and granulocyte infiltration described in a recently published study identifying six lesion types.^10^ In contrast, the second case only demonstrated pathological findings compatible with the chronic NMOSD lesions in post-mortem cases, and the differential diagnosis of NMOSD based solely on the pathological findings is challenging because of the similarity of chronic NMO lesions to another demyelinating diseases such as MS. The elevated expression of AQP4 in the demyelinated lesions is indicative of reactive fibrous astrogliosis that can also be observed in MS, whereas speckled necrotic spots with decreased AQP4 staining and vascular hyalinization are consistent with astrocyte dystrophy found in NMO. This sample may have been taken from the periphery of the active lesion, or the biopsied area may have been affected by a previous attack.

Although we do not under any circumstances recommend using spinal cord biopsy as a substitute for AQP4-antibody testing to diagnose NMOSD, the AQP4-antibody result may be false negative with some assays and the diagnosis may be arrived at incidentally during the work-up of spinal tumors. NMOSD should be suspected in biopsies with features such as cystic changes with vascular wall thickening and astrocyte rarefaction. The findings can range from the very acute to chronic inactive lesions depending on the evolutionary stage of the biopsied area. Nevertheless, additional immunohistochemistry staining is required to identify other features compatible with the typical pathological findings described in NMOSD.

## References

[1] Trebst C Raab P Voss EV Longitudinal extensive transverse myelitis—it’s not all neuromyelitis optica. Nat Rev Neurol 2011; 7: 688–698. 22045269 10.1038/nrneurol.2011.176

[2] Wingerchuk DM Lennon VA Lucchinetti CF The spectrum of neuromyelitis optica. Lancet Neurol 2007; 6: 805–815. 17706564 10.1016/S1474-4422(07)70216-8

[3] Sato DK Nakashima I Takahashi T Aquaporin-4 antibody-positive cases beyond current diagnostic criteria for NMO spectrum disorders. Neurology 2013; 80: 2210–2216. 23677744 10.1212/WNL.0b013e318296ea08

[4] Sato D Callegaro D Lana-Peixoto MA Treatment of neuromyelitis optica: An evidence based review. Arq Neuropsiquiatr 2012; 70: 59–66. 22218475 10.1590/s0004-282x2012000100012

[5] Dellavance A Alvarenga RR Rodrigues SH Anti-aquaporin-4 antibodies in the context of assorted immune-mediated diseases. Eur J Neurol 2012; 19: 248–252. 21771203 10.1111/j.1468-1331.2011.03479.x

[6] Lucchinetti CF Mandler RN McGavern D A role for humoral mechanisms in the pathogenesis of Devic’s neuromyelitis optica. Brain 2002; 125: 1450–1461. 12076996 10.1093/brain/awf151 PMC5444467

[7] Misu T Fujihara K Kakita A Loss of aquaporin 4 in lesions of neuromyelitis optica: Distinction from multiple sclerosis. Brain 2007; 130: 1224–1234. 17405762 10.1093/brain/awm047

[8] Habek M Adamec I Brinar VV Spinal cord tumor versus transverse myelitis. Spine J 2011; 11: 1143–1145. 22082692 10.1016/j.spinee.2011.10.012

[9] Fujihara K Misu T Nakashima I Neuromyelitis optica should be classified as an astrocytopathic disease rather than a demyelinating disease. Clin Exp Neuroimmunol 2012; 3: 58–73.

[10] Misu T Höftberger R Fujihara K Presence of six different lesion types suggests diverse mechanisms of tissue injury in neuromyelitis optica. Acta Neuropathol 2013; 125: 815–827. 23579868 10.1007/s00401-013-1116-7 PMC3661909

